# Charcot-Marie-Tooth: From Molecules to Therapy

**DOI:** 10.3390/ijms20143419

**Published:** 2019-07-12

**Authors:** Jonathan Morena, Anirudh Gupta, J. Chad Hoyle

**Affiliations:** 1 Department of Neurology, The Ohio State University, Columbus, OH 43210, USA; 2 Department of Neurology, Division of Neuromuscular Disorders, The Ohio State University, Wexner Medical Center, Columbus, OH 43210, USA

**Keywords:** Charcot-Marie-Tooth, hereditary neuropathy, molecular therapy, gene therapy

## Abstract

Charcot-Marie-Tooth (CMT) is the most prevalent category of inherited neuropathy. The most common inheritance pattern is autosomal dominant, though there also are X-linked and autosomal recessive subtypes. In addition to a variety of inheritance patterns, there are a myriad of genes associated with CMT, reflecting the heterogeneity of this disorder. Next generation sequencing (NGS) has expanded and simplified the diagnostic yield of genes/molecules underlying and/or associated with CMT, which is of paramount importance in providing a substrate for current and future targeted disease-modifying treatment options. Considerable research attention for disease-modifying therapy has been geared towards the most commonly encountered genetic mutations (*PMP22*, *GJB1*, *MPZ*, and *MFN2*). In this review, we highlight the clinical background, molecular understanding, and therapeutic investigations of these CMT subtypes, while also discussing therapeutic research pertinent to the remaining less common CMT subtypes.

## 1. Background

Hereditary (genetic) neuropathies can either occur as part of a multisystem disease or with neuropathy as the primary feature (primary hereditary neuropathies). Phenotypic classifications on the far ends of the spectrum of primary genetic neuropathies include the hereditary motor neuropathies (HMN) with minimal to no sensory involvement and the hereditary sensory neuropathies (HSN) with significant sensory involvement (may have skin ulcerations, due to the significance of the sensory involvement, or autonomic involvement). Charcot-Marie-Tooth (CMT) disease indicates a genetically heterogeneous group of primary genetic neuropathies classically with sensory and motor involvement, termed hereditary sensory and motor neuropathy (HSMN). Examples of genetic multisystem disorders in which neuropathies are only one aspect of the syndrome include Friedreich ataxia and other inherited ataxias; familial amyloidosis; lysosomal storage disease, such as some of the leukodystrophies and Fabry disease; lipid disorders, such as Tangier disease and abetaliproteinemia; peroxisomal disease, i.e., Refsum disease; porphyria; and several mitochondrial disorders [1,2].

CMT is globally the most common inherited disorder of peripheral nerves (neuropathy) with a prevalence of 1:2500 [3]. In eastern Akershus County, Norway, prevalence is twice as high (1:1214), whereas in Japan, for example, there is a lower reported prevalence of 1:9200 [4]. This may be caused by different methods of ascertainment or founder effects. The genetic heterogeneity manifests in different patterns of inheritance (autosomal dominant, autosomal recessive, X-linked), as well as distinct electrophysiological classes (axonal, demyelinating, intermediate). Autosomal dominant inheritance is the most frequent pattern. Of note, sporadic cases of hereditary neuropathies resulting from de novo mutations have been described (at least 10% of cases of CMT1A) [5].

Currently, >90 distinct genetic variations have been implicated in causing or contributing to the clinical picture of these neuropathies [2]. They have been linked to diverse molecular pathomechanisms, which either involve protein synthesis and posttranslational processing (abnormal mRNA processing, dysfunction of endosomal sorting and signaling, proteasomal/protein aggregation abnormalities, aberrant myelin assembly), intracellular trafficking (axonal transport/cytoskeletal abnormalities), dysfunction of ion channels (channelopathies), or mitochondrial dysfunction [6,7]. Though there are a myriad of gene associations and pathophysiologic mechanisms, four genes comprise over 90% of genetically confirmed cases of CMT (*PMP22*, *MPZ*, *GJB1*, and *MFN2* genes) (See Table 1) [8].

CMT patients typically present clinically/phenotypically (despite their genetic heterogeneity) with an indolent, length-dependent, sensorimotor polyneuropathy. The slowly progressive nature of the condition (as opposed to an immune-mediated etiology) and noticeable length-dependent weakness/atrophy (rather than a predominantly stocking glove sensory loss with minimal to no weakness more typical of common metabolic-toxic neuropathies) are clinical clues to CMT in addition to uncovering any suggestive family history. The majority of patients also have pes cavus as a hallmark feature with smaller percentages having scoliosis, hip dysplasia, restless legs syndrome, tremor, or hearing loss [9,10,11,12,13,14,15,16,17].

As noted, electromyography helps characterize the polyneuropathy in CMT by reflecting different degrees of myelin versus axonal pathophysiology. The categorical degrees are demyelinating, axonal, and intermediate. These categories are classified based on motor nerve conduction velocities in the upper extremities. Slow nerve conductions imply demyelinating pathology. A protocol defined demyelination as having a conduction velocity range of 15–35 m/s (severe demyelination less than or equal to 15 m/s); axonal as greater than 45 m/s; and intermediate conduction velocity range as more than 35 and up to 45 m/s [8]. These conduction velocity parameters, as well as the pattern of inheritance help stratify CMT into major categories defined in the following way: CMT1 as demyelinating subtype (autosomal dominant); CMT2 as axonal subtype (autosomal dominant or recessive); CMTX with intermediate conduction velocities (most classically X-linked, though there are autosomal dominant and recessive intermediate variants); and CMT4 again with a demyelinating subtype (but autosomal recessive). Prior nomenclature has labeled CMT3 as the infantile (Dejerine Sottas) or birth onset (congenital hypomyelinating neuropathy) CMT neuropathies.

This electrophysiological characterization (as well as taking into account gene mutation prevalence) helped facilitate an algorithmic approach to targeted genetic testing and diagnosis, although more recently next generation sequencing (NGS) has become a favored cost effective option to test the full range of CMT genes simultaneously [8,18,19]. Specific genes can be suspected if certain unusual clinical features are encountered- tonic pupils in CMT 1B (*MPZ*); central nervous system (CNS) stroke-like events/white matter changes or a split hand, characterized by disproportionate motor loss of the thenar eminence of the hand with CMT1X (*GJB1*); optic atrophy in CMT2A (*MFN2*), or vocal cord/diaphragm paralysis in CMT2C (*TRPV4*) (See Table 1) [20,21,22,23,24,25,26,27,28]. Nevertheless, despite these helpful clinical correlates, targeted strategies are being replaced by next generation sequencing as the most efficient and cost-effective strategy for diagnosis. With high suspicion for CMT1A (i.e., motor conduction velocities in the upper extremities of 15–35 m/s) the targeted yield could still favor screening for *PMP22* (*Peripheral Myelin Protein 22*) duplication first, but generally proceeding initially to full panel testing is becoming the preferred diagnostic approach [29].

Historically, at least 60% of CMT patients could be genetically confirmed with targeted testing (higher yield for CMT1 than CMT2), and NGS panel testing is expanding this diagnostic yield [8,30,31,32]. In patients who are negative on panel testing, whole exome sequencing (WES)/whole genome sequencing (WGS) can be pursued. However, with the advent of the ability to cast a wide net in genetic screening, it is critical to not overinterpret variants of undetermined significance (VUS) as causative. As a sizable minority of CMT patients have no known gene mutation, continued scholarly collaboration with molecular biologists will be instrumental in uncovering the remaining holes in diagnostic yield.

There are currently no effective pharmacologic treatments for CMT, limiting historic treatment to supportive care. Ankle-foot orthoses (AFOs) are externally applied devices that have typically been prescribed for foot drop and can improve balance and gait in patients with CMT [33]. AFOs aid in ankle dorsiflexion in patients with CMT and ameliorate the need for compensatory hip action to overcome the foot drop [34]. Anterior elastic AFOs have specifically been shown in CMT1A patients to enhance walking economy, allowing patients to expend less energy per unit of walking [35].

Physical rehabilitation and exercise are other supportive care measures that have been utilized and studied. Resistance and aerobic exercise have shown benefits in CMT [36,37,38,39,40]. Of note, overwork weakness (weakness increased by exercise, work, or daily activities) has been hypothesized to be present in CMT, due to a study that demonstrated dominant hand muscles are weaker in patients with CMT. However, this was not replicated in subsequent studies, and physical activity and rehabilitation remain encouraged for patients with CMT [40,41,42,43]. In addition to exercise, creatine supplementation has been tested in CMT; however, with inconsistent results [36,44,45].

Intermittent fasting has been studied in the Trembler J (Tr-J) mouse model of CMT1A. One study showed that affected mice on a five-month long intermittent fast had increased expression of myelin proteins, a thicker myelin sheath, less aberrant Schwann cell proliferation and a decrease in PMP22 protein aggregates. The intermittent fasting regimen enhanced endogenous protective mechanisms, such as the chaperone and autophagy-lysosomal pathways, and prevented the inflammatory and degenerative changes associated with neuropathy. This resulted in improved locomotor performance and increased grip strength in the mice [46,47,48,49].

The ultimate path forward to yield the most significant disease modifying treatments will most likely center around addressing the underlying molecular pathogenesis with targeted molecular therapy. Given the vast genetic heterogeneity of CMT, therapeutic investigations geared at pathophysiologic mechanisms have been weighted toward, but not been limited to, studying the more common gene mutations. This review will highlight progress in the four most common mutations underlying CMT, while also discussing an evolving understanding of other therapeutic strategies related to an expanding understanding of the pathogenic mechanisms that underlie this heterogeneous disorder as a whole.

## 2. CMT1A

CMT1A is caused by a 1.4 Mb duplication of the *PMP22* gene (encoding peripheral myelin protein 22 kD) and is the most common hereditary neuropathy [8]. Overexpression of PMP22 causes a demyelinating neuropathy [50,51]. Clinically, patients typically present with the classic CMT phenotype of distal weakness/atrophy, sensory loss, and high arched feet. The majority of patients have reduced or absent reflexes in both the arms and legs [52]. The pattern of weakness is almost always length-dependent [52,53]. The age of onset is in the first decade in 75% of patients with later ages of onset in a minority of patients. A more severe variant that occurs during infancy or early childhood is called Roussy-Levy syndrome. This syndrome is typified by an upper limb postural tremor and represents less than 15% of cases. The mean upper limb motor conduction velocity with PMP22 duplication is 19.9 m/s with a range of 5–34 m/s [53]. The conduction slowing is typically uniform and symmetrical (*MPZ*, *GJB1*, *Fig4*, and *LITAF* mutations, on the other hand, have more characteristically been reported to mimic an acquired immune disorder at times on the nerve conductions) [20,54,55,56,57]. Nerve biopsy demonstrates demyelination with onion bulb formation [53].

Strategies to downregulate *PMP22* gene expression have been a logical extension of research efforts. Ascorbic acid was one of these first therapies evaluated for CMT, demonstrating promise in a mouse model [58]. Administration of progesterone receptor antagonists has also reduced overexpression of PMP22 and improved axonal integrity in a transgenic rat model [59]. More recently, non-viral delivery of small interfering RNA (siRNA) in Tr-J mice suppressed the *PMP22* mutant allele, enhanced myelination of Schwann cells and ameliorated the demyelinating phenotype [60].

Unfortunately, so far successful translation to human trials has been discouraging. Human clinical trials evaluating ascorbic acid have not shown significant beneficial effects [61,62,63]. These trials highlight the difficulty of translating preclinical to clinical success. Disorders with a very indolent progression make differentiation from control groups more challenging (in addition to necessarily increasing trial duration). Additionally, modulation of PMP22 protein levels have caveats- levels vary significantly over time in patients, and the absolute value at a given time point is not predictive of phenotype. It might be that stabilizing excessive fluctuations of PMP22 levels rather than just downregulating the absolute value is necessary for disease modification or that modulating *PMP22* expression is only effective if initiated in an early, time limited window of the disease course [64,65]. Despite disappointments in early clinical trials, given the pathophysiology of *PMP22* overexpression as a fundamental abnormality in CMT1A, continuing efforts to explore and refine treatments that alter abnormal PMP22 processing remain an active area of research interest.

Further promising research has discovered a combination of already marketed and known medications- baclofen, sorbitol and naltrexone (PXT3003) have been found to act synergistically in improving myelination and down-regulating *PMP22* mRNA expression in a transgenic rat model [66]. PXT3003 has also been shown to improve the misbalanced down-stream PI3K-AKT/MEK-ERK signaling pathways that have been implicated in regulating Schwann cell differentiation. Short-term treatment with PXT3003 during early development has been shown to ameliorate the long-term phenotypical manifestations in CMT1A rats. Therefore, if translated to CMT1A patients, treatment should be initiated as early as possible in order to maximize the impact on disease manifestation [67]. PXT3003 has been shown to be safe and tolerable in a phase II trial in CMT1A patients with a continued rationale for further studies [68,69]. There is currently a Phase III trial in the process to evaluate the long-term safety and tolerability (ClinicalTrials.gov NCT03023540). 

Antisense oligonucleotides (ASOs) have been used for a variety of disorders and have been translated into clinical trials showing that they can safely be used in humans [70]. Examples of recent clinical successes include nusinersen for spinal muscular atrophy, as well as inotersen for transthyretin (TTR) hereditary amyloidosis [71,72,73]. ASOs can suppress PMP22 mRNA levels in a dose-dependent fashion and result in improvement in cellular, functional, and electrophysiological measures in the rodent model of CMT1A [74].

A divergent therapeutic pathway to investigate involves modification of lipid metabolism. It represents an approach in CMT1A that is potentially easily clinically translatable. Myelinating Schwann cells in a rat model of CMT1A exhibited reduced transcription of genes for myelin lipid biosynthesis. Lipid incorporation into myelin was therefore reduced. Phosphatidylcholine and Phosphatidylethanolamine in the diet have shown to alter the myelination deficit of affected Schwann cells. This lipid supplementation bypassed the inefficient expression of genes for lipid synthesis in PMP22 transgenic Schwann cells and resulted in improved myelin biosynthesis and reduced neuropathic symptoms in a CMT1A rat model. The therapeutic effect was observed not only during postnatal development, but also in older CMT rats, suggesting the potential for disease modification across the entire lifespan [75]. Dietary phospholipids have been tested in clinical trials and have shown no significant side effects, highlighting the ease of clinical translation if this is an effective therapeutic route [76].

In addition, aberrant Schwann cell differentiation has been found to be a potential factor in the pathophysiology of CMT1A. One study utilized human induced pluripotent stem cell (hiPSC) lines from two CMT1A patients as an in vitro cell model. *PMP22* duplication led to a developmental switch of Schwann cell differentiation toward endoneurial fibroblast-like cells instead of neural crest cells. This may cause excessive cell proliferation, defects in and the ability for myelination, as well as dysregulation of cholesterol/lipid biosynthesis and transportation [77]. These findings related to the pathophysiology of CMT1A could lead to additional therapeutic opportunities.

## 3. CMTX1

CMTX1 is the second most common form of hereditary motor and sensory neuropathy and has an X-linked dominant inheritance pattern. A family history of disease with no male-to-male transmission and motor conduction responses with an intermediate velocity raise suspicion for this diagnosis. Clinically, patients have a mild to moderate CMT phenotype with males being more clinically affected than females. Motor conduction velocities typically range between 30–40 m/s in males and between 30 m/s to normal in females. Active axonal degeneration on needle electrode examination is rare [78]. Asymmetric and non-homogenous conductions can mimic an acquired process electrophysiologically with CMTX1, rather than just the classic uniform slowing more typical of CMT [79]. As mentioned, CNS stroke-like events or white matter changes have been associated with CMTX [21,22,23]. A ‘split hand,’ characterized by disproportionate motor loss of the thenar eminence, may be a clinical clue to this disorder, as well (in one study out of 32 family members, all affected adult patients had predominant involvement of the thenar muscles) [28].

CMTX1 is caused by a mutation in *GJB1* (*gap junction protein beta 1*), a gene that encodes the gap junction protein connexin 32 (Cx32). The gap junctions formed by Cx32 play an important role in the homeostasis of myelinated axons. Loss of Cx32 in myelinating Schwann cells has been shown to cause a demyelinating neuropathy [80]. It is also likely that mutations in Cx32 result in mitotic instability through calcium/calmodulin-dependent kinase type II (CamKII) overexpression [81]. CAMKII has a critical role in regulating oligodendrocyte maturation and CNS myelination [82]. Treatment with CamKII inhibitors has resulted in a partial rescue of the cellular phenotype, including partial restoration of connexons in sciatic nerves and improvement in the phenotype of transgenic mice [81]. The fibroblasts of CMTX patients have been shown to display a similar phenotype as the fibroblasts of mouse models that can be corrected by CAMKII inhibitors. This suggests that the success of CMAKII inhibitors in mouse models is potentially transferable to humans [83].

*GJB1* mutations are most commonly caused by loss-of-function mutations. This supports the concept that gene replacement could be beneficial. However, counterproductive interactions between a delivered wild type *Cx32* gene and some specific mutant *Cx32* forms may occur. In those particular mutations, the virally delivered gene therapy does not traffic normally to deliver therapy optimally. Thus, screening for mutant-wild type interaction will be an important caveat to gene replacement therapy strategies [84]. In the proper context, gene replacement therapy might lead to a viable treatment option. Intraneural gene delivery using a lentiviral vector has been shown to result in efficient gene expression in Schwann cells with improvements in nerve pathology [85]. Additionally, intrathecal gene therapy in mutant mouse models with non-interfering CMTX1 mutations altered the peripheral nervous system pathology, improving the functional and morphological properties of the demyelinating neuropathy [86]. Future studies will need to assess the viability of this approach in human clinical trials, as well as innovate solutions to overcome the effect of specific gene mutations that could impair correct intracellular trafficking of virally delivered therapy. Gene replacement is a very promising technology, particularly given the recent clinical success of single dose intrathecal gene replacement therapy for spinal muscular atrophy [87].

## 4. CMT1B

CMT1B is the next most common HSMN and caused by mutations of *MPZ* (*myelin protein zero*), which is the major protein constituent of peripheral myelin. Clinically, a classic CMT phenotype may be seen, though more commonly early- or late-onset clinical presentations are associated with mutations of *MPZ*. The early-onset variant causes the severe neuropathy of infancy, denoted as Dejerine-Sottas disease, with significant clinical involvement and severe demyelination. Conversely, the adult-onset variant with signs and symptoms that start around the age of 40 presents with a milder phenotype and ‘CMT2′ type normal conduction velocities [88]. A patchy neuropathy with conduction block and tonic pupils has been reported with *MPZ* mutations, as well, highlighting the genetic variability that might also mimic an inflammatory cause of neuropathy [20].

Frameshift mutations in *MPZ* can cause intracellular accumulation of mutant proteins in the endoplasmic reticulum (ER) inducing apoptosis and subsequent neuropathy. Curcumin, a chemical compound derived from turmeric, releases the ER retained MPZ mutants into the cytoplasm and results in a significant decrease in apoptosis [89]. Curcumin has had positive results in R98C mice, a model of CMT1B. Curcumin given to these mice improved neuropathy by decreasing ER stress, reducing activation of the unfolded protein response, and promoting Schwann cell differentiation [90,91]. Of interest, curcumin has also been shown to reduce apoptosis in PMP22 mutations through a similar mechanism. When used in the Tr-J mouse model, a PMP22 mutant mouse model to study CMT1A, curcumin reduced neuropathy in a dose-dependent manner [92].

When mutant proteins are retained in the ER, the unfolded protein response (UPR) is activated. This is an adaptive and protective process to relieve stress from the misfolded proteins. However, when cells are in chronic stress, such as in CMT1B, the UPR becomes inadequate, and it activates apoptotic pathways resulting in cell death or signaling that alter the normal phenotype. A large number of *MPZ* mutations activate the UPR and cause CMT1B, and these mutations may be susceptible to future therapies [93]. Sephin1 (through selective inhibition of a phosphatase regulatory subunit) prolonged the UPR and prevented the molecular, morphological and motor defects of the neuropathy of MPZ mutant mice [94].

## 5. CMT2A

CMT2A accounts for upward of 30% of CMT2 patients, making it the most common variant in that cohort [30]. Patients that present with axonal range motor conduction velocities may actually more commonly have CMTX1, though (See Table 1). Clinically, patients most commonly present with a more severe and earlier onset phenotype than classic CMT, though milder, late-onset phenotypes may also be encountered as part of a bimodal distribution of the genetic phenotype [95,96]. Optic atrophy and vocal cord palsy have been described in both the early-onset, severe phenotype, as well as the late-onset, milder phenotype. Additionally, CNS involvement may be seen in CMT2A, including spinal cord abnormalities in 26% (spinal cord atrophy or hydromyelia; rarely with brisk reflexes on the exam) or brain involvement, such as periventricular, brainstem, cerebellar white matter changes or, cortical atrophy [24].

CMT2A is caused by mutations in *MFN2* (*Mitofusin-2*) [97]. MFN2 is also involved in other aspects of mitochondrial metabolism, as well as cell signaling and apoptosis [98]. Coenzyme Q10 (CoQ10) supplementation plays a role in mitochondrial health as part of the electron transport chain and is involved in energy production. It also plays a role as an antioxidant. Coenzyme Q10 supplementation was associated with improvement in visual impairment in a patient with hereditary motor sensory neuropathy type VI (i.e., CMT2A with optic atrophy). Given this subtype’s pathomechanism of mitochondrial dysfunction through the *MFN2* mutation, coenzyme Q10 supplementation may have ameliorated the phenotype; further targeted studies are warranted [99]. A promising upcoming therapeutic target for CMT2A involves mitofusin agonists, more directly addressing the underlying pathophysiology of *MFN2* mutations. Mitofusin agonists have been shown to normalize mitochondrial trafficking within sciatic nerves of MFN2 Thr105Met mice, showing a promising therapeutic approach for CMT2A [100].

## 6. Other Therapeutic Avenues

Outside of the four main genes causing CMT, there are common mechanisms and other genes that are being researched to help develop other therapeutic options. These include histone deacetylase 6 (HDAC6) inhibition, stem cells, Neurotrophin-3 (NT-3), Neuregulin-1 Type III (Nrg1 TIII), tumor necrosis factor-α converting enzyme (TACE) modulation, colony-stimulating factor 1 receptor (CSF1R) inhibition, and follistatin based therapy.

Mutations in heat-shock protein gene 1 (*HSPB1*) can have a relation to axonal forms of CMT. Mutant HSPB1 mice have decreased acetylated alpha-tubulin and severe axonal transport deficits. Inhibition of HDAC6, a histone deacetylase, has been shown to increase acetylated alpha-tubulin and correct the axonal transport defects. This resulted in rescuing the CMT2 phenotype of mutant HSPB1 mice [101]. To translate these results into clinical application, potent and selective HDAC6 inhibitors are being explored. ACY-738, ACY-775, and ACY-1215 have demonstrated to be both potent and selective HDAC6 inhibitors, and they functionally improved the motor and sensory deficits in a CMT2F mouse model. ACY-1215 has been shown to be safe and well tolerated in clinical trials for cancer [102]. Furthermore, HDAC6 inhibition has been shown to partially restore nerve conduction and motor behavior in mutant *Gars* mice with reduced levels of acetylated alpha-tubulin. This suggests that decreased acetylated alpha-tubulin may represent a common pathomechanism and that HDAC6 inhibitors may have therapeutic potential for some other forms of axonal CMT [103].

Stem cell research and neurotrophic factors, including NT-3, have shown promising results in Tr-J mice. One study investigated the potential of Schwann-like cells differentiated from human tonsil-derived stem cells. These Schwann-like cells were transplanted into the caudal thigh of Tr-J mice and resulted in neuromuscular regeneration [104]. Stem cells also secrete neurotrophic factors, which promote axonal growth and remyelination [105]. NT-3 has improved axonal regeneration and myelination in Tr-J mice. It has also shown efficacy in CMT1A patients [106]. Long-term treatment was not possible, due to its short half-life and lack of availability. However, NT-3 gene therapy through an adeno-associated virus vector achieved sustained NT-3 levels [107]. NT-3 has also more recently been shown to increase muscle fiber diameter in Tr-J mice through direct activation of the mTOR pathway. This may allow for the development of new therapeutic strategies regulating the mTOR pathway and combination strategies with NT-3 [108].

Schwann cells in rodent models of CMT1A acquire a persistent differentiation defect during early postnatal development. This is caused by reduced activity of PI3K-AKT signaling and increased activity of the MEK-ERK pathway. Early recombinant human Neuregulin 1 (Nrg1) therapy induces PI3K-AKT activity and balances the ratio of PI3K-AKT/MEK-ERK signaling. This treatment improves Schwann cell differentiation and is neuroprotective [109]. Nrg1 type III is an essential signal for peripheral myelination, and the amount of axonal Nrg1 determines whether a nerve becomes myelinated [110]. Nrg1 type III is inhibited by tumor necrosis factor-α converting enzyme (TACE), and TACE subsequently inhibits myelination. Mutant mice lacking TACE are hypermyelinated, and their phenotype resembles Nrg1 overexpressing mice. Niaspan enhances TACE and ameliorates the neuropathy in a mouse model of CMT4B1 with myelin outfoldings by reducing the hypermyelination [111]. Stimulating Nrg1 type III by suppressing TACE improves neuropathy in a mouse model of CMT1B [112]. Thus, regulation of Nrg1 TIII is a potential candidate for a general therapeutic approach for CMT.

Low-grade inflammation from phagocytizing macrophages contributes to the neuropathic phenotype in mouse models for CMT1A, CMT1B and CMTX1 [113]. An important macrophage activator is colony-stimulating factor 1 (CSF1). It has been shown that inhibition of CSF1R leads to a decline in nerve macrophages. In CMTX1 and CMT1B mice, CSF1R inhibition was a safe and effective treatment option resulting in functional and structural benefits of peripheral nerves [114].

In addition, a more general approach of increasing muscle volume is currently being explored involving ACE-083. ACE-083 is a locally acting follistatin-based molecule that has been shown to increase skeletal muscle mass and force through inhibition of myostatin and other muscle regulators. A Phase 1 trial showed that it was well tolerated and led to increased muscle growth in the rectus femoris and tibialis anterior muscles [115]. A Phase 2 trial is now underway to evaluate the safety, tolerability, bioavailability and effectiveness in patients with CMT1 and CMTX (ClinicalTrials.gov NCT03124459).

See Table 2 for a summary of treatment investigations in CMT.

## 7. Conclusions

There has been a significant advancement, as well as much ongoing and upcoming research in the diverse disorder of CMT. Molecular understanding is expanding, and targeted therapy with the potential for disease modification appears closer on the horizon. Challenges remain, though. Molecular mechanisms underlying CMT are diverse and eliciting a specific therapy for each subtype will require much further study. Additionally, given the diversity of pathophysiology, there are limitations to discovering a robust therapy that translates across the multiple subtypes of CMT. Translating molecular studies to clinical trials is also hindered by the challenge of eliciting a clear effect size with very slow disease progression as a control. Despite these challenges, progress is being made, and future therapeutic potential is promising, as has been outlined. Improving biomarkers, as well as natural history data (NCT01193075) may help facilitate clinical trials of these upcoming disease-modifying treatments. Advancements in gene therapy are having implications for the field of neurology as a whole and have implications for CMT, as well. Gene replacement therapy is germane to mutations with loss-of-function and is being explored in animal models with CMT1X and CMT4C [86,122,123]. Other gene therapy strategies for a wide variety of mechanisms in CMT are likely to continue to expand, as well. Advancement in molecular therapeutic potential is yielding hope for future disease modifying treatments for CMT.

## Figures and Tables

**Table 1:** Frequency * of the most common genetically Charcot-Marie-Tooth (CMT) mutations and their clinical associations.

CMT Subtype (Gene Involved)	Inheritance Pattern, Conduction Velocities, Clinical Features	Frequency of Occurrence in Genetically Confirmed CMT Cases
CMT1A (*PMP22* duplication)	Autosomal dominant, most common subtype of CMT overall, most common demyelinating form (89% yield with CMT-like phenotype and motor conduction velocities between 15–35 m/s in the upper extremities)	60.5%
CMTX1 (*GJB1*)	X-linked, intermediate motor conduction velocities, stroke-like episodes or white matter changes, split hand syndrome	16.7%
CMT1B (*MPZ*)	Autosomal dominant, demyelinating motor conduction velocities, tonic pupils	9.4%
CMT2A (*MFN2*)	Autosomal dominant, most common form of CMT2, axonal motor conduction velocities, optic atrophy	4.4%

* Frequency distribution adapted from Saporta et al. 2011 [8] (excluding HNPP—hereditary neuropathy with liability to pressure palsies).

**Table 2:** Therapy investigations CMT.

PMP22	GJB1	MPZ	MFN2	Other
PXT3003 (3) [66,67,68,69] *ClinicalTrials.gov* NCT03023540	CAMKII inhibitors (p) [81,82,83,116]	Curcumin (p) [89,90,92]	Coenzyme Q10 [99]	Follistatin-based therapy (2) [115] *ClinicalTrials.gov* NCT03124459
Vitamin C (P, 1–3) [6,58,61,62,63,117,118,119,120]	Cx32 gene therapy (p) [85,86]	Sephin 1 (p) [94]	Mitofusion agonists (p) [100]	Stem cell research (p) [104,105]
Progesterone Antagonists (p) [59,121]				Gene therapy (p) [85,86,107,108,122,123]
siRNA (p) [60]				HDAC6 Inhibition (p) [101,102,103]
Antisense Oligonucleotides(p) [74]				NT-3 (p) [106,107,108]
Lipid supplementation (p) [75]				Nrg-1Type III (p) [109,110]
Schwann cell differentiation (p) [50,77]				TACE modulation (p) [111,112]
Curcumin (p) [89,90,92]				CSF1R inhibition (p) [114]
				Intermittent Fasting (p) [46]

(p) = Preclinical; (0) = Phase 0 clinical trial; (1) = Phase 1; (2) = Phase 2; (3) = Phase 3.
